# Supplementary material for: SARS-CoV-2 Receptor Angiotensin I-Converting Enzyme Type 2 (ACE2) Is Expressed in Human Pancreatic β-Cells and in the Human Pancreas Microvasculature
Source: Front Endocrinol (Lausanne). 2020 Nov 13;11:596898. doi: 10.3389/fendo.2020.596898 (PMC7691425; doi:10.3389/fendo.2020.596898)
Supplement: Supplementary file 1 [file DataSheet_1.pdf]

SUPPLEMENTARY MATERIAL

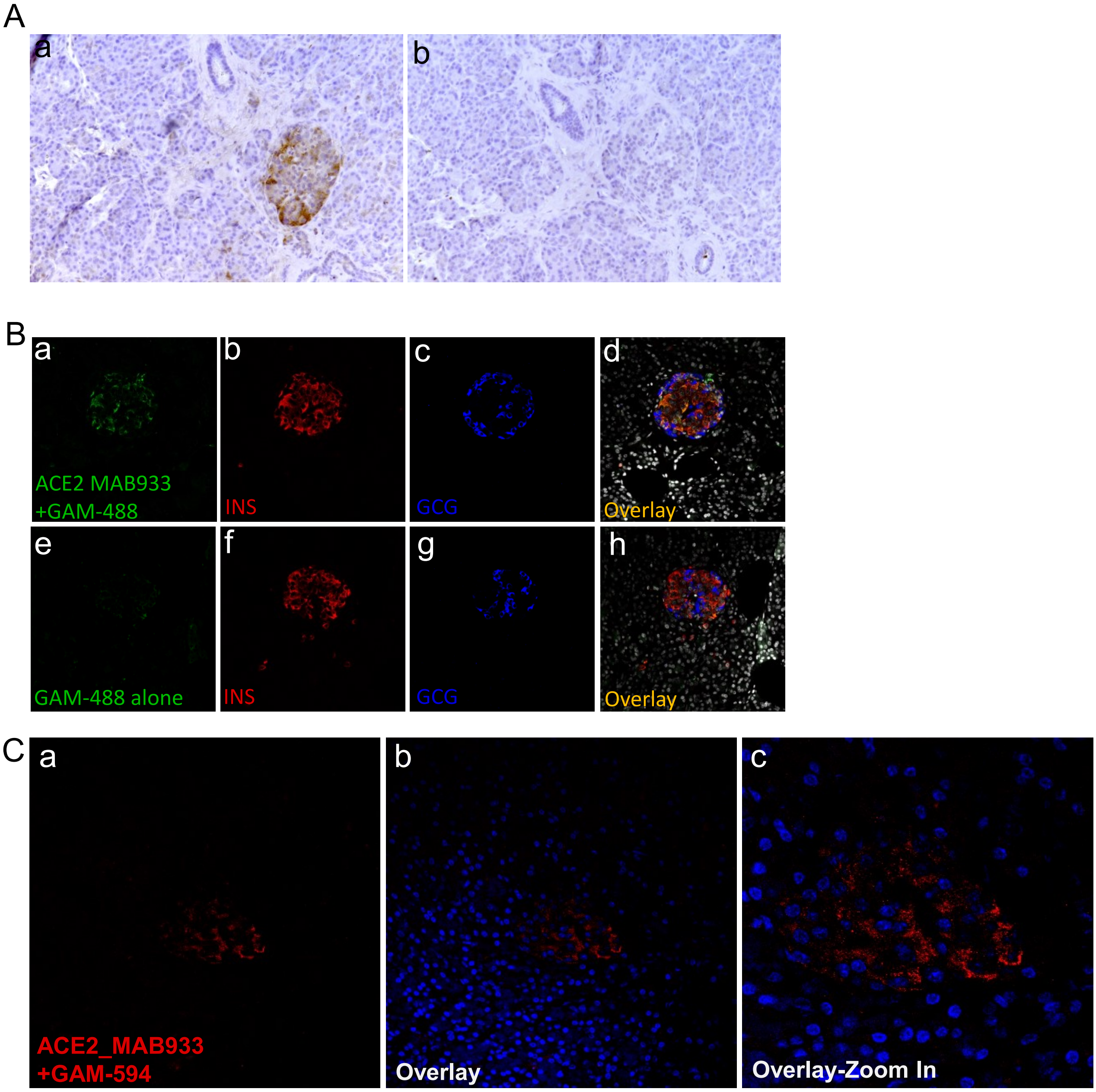

Figure S1. ACE2 negative control staining in human pancreas. (**A**) Immunohistochemistry of a Formalin-Fixed Paraffin Embedded (FFPE) pancreatic section from an adult non-diabetic multiorgan donor (id: #210518) using MAB933 anti-ACE2 antibody (panel-a) or secondary antibody-only (panel-b). (**B**) Triple Immunofluorescence of a FFPE pancreatic section from an adult-non diabetic multiorgan donor (id: 210518) using MAB933 anti-ACE2 antibody (panel-a) or fluorescently-labeled Goat Anti Mouse (GAM) secondary antibody-only as negative control for ACE2 (panel-b). (**C**) ACE2 immunofluorescent staining in FFPE pancreatic section from an adult-non diabetic multiorgan donor (id: 210518) using MAB933 anti-ACE2 antibody (panel-a) with another fluorescently labeled Goat Anti Mouse-594, in order to exclude background staining artifacts due to the previously adopted ACE2 fluorescent detection system.

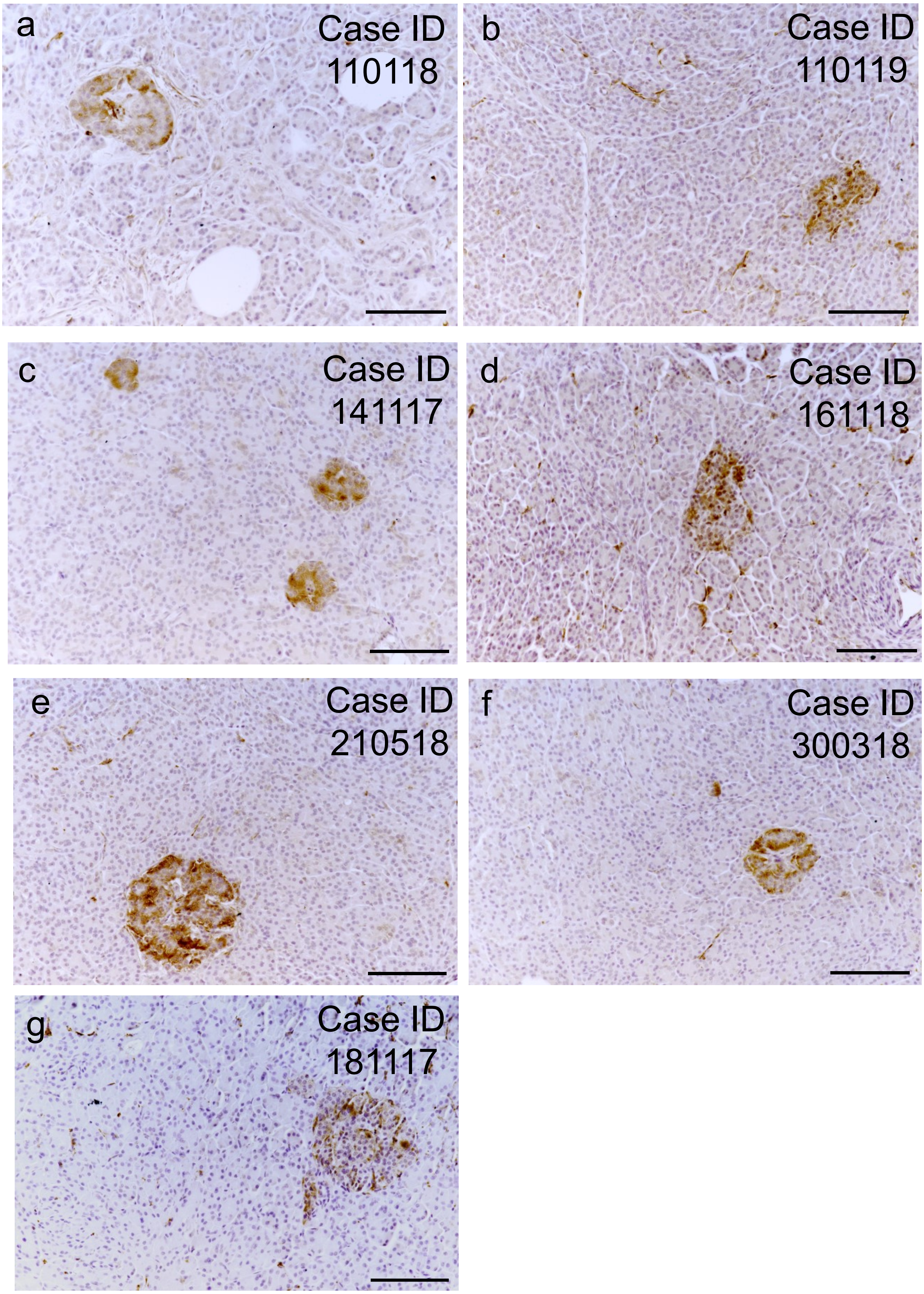

Figure S2. Representative images of all cases showing expression of ACE2 (MAB933) in human pancreatic islets. Representative images of ACE2 IHC staining in human pancreatic FFPE sections obtained from seven different non-diabetic multiorgan donors. Scale bar: 100 µm.

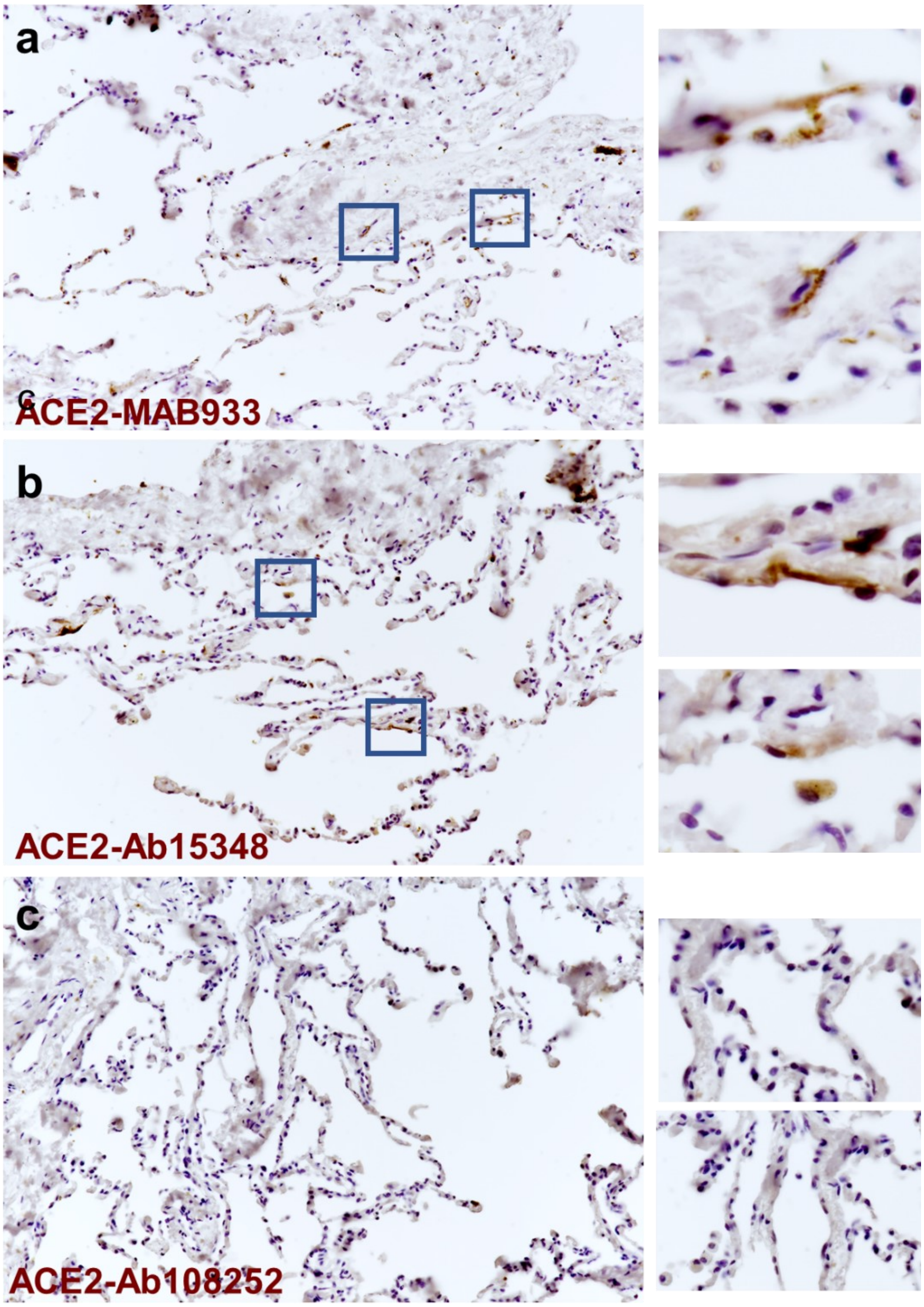

Figure S3. **ACE2 staining positive control**. Anti-ACE2 MAB933 (panel-a), Ab15348 (panel-b) and Ab108252 (panel-c) antibodies immunohistochemistry in FFPE lung tissue sections. Zoom-in insets are shown on the right, adjacent to each main related image.

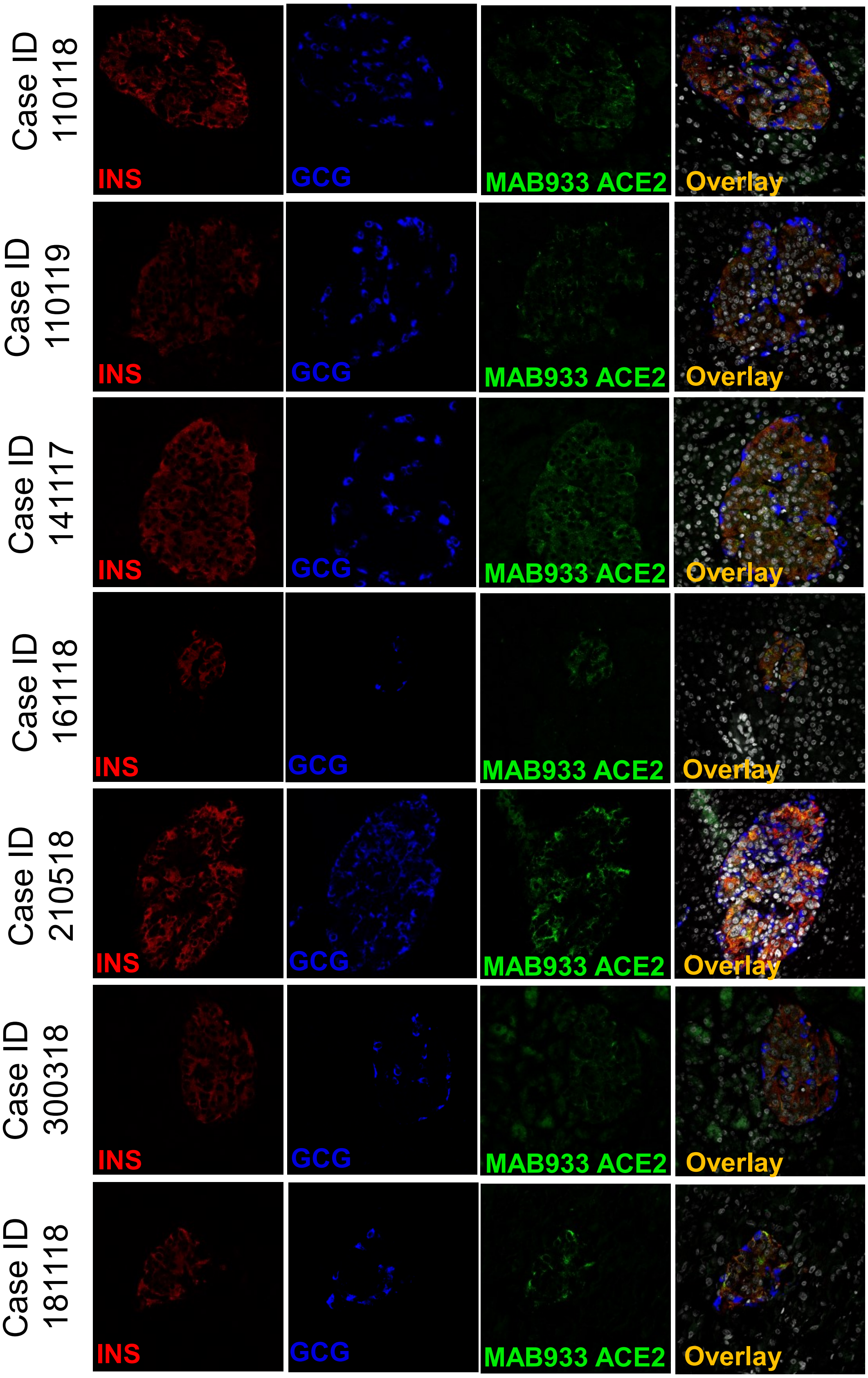

Figure S4. ACE2 MAB933 immunofluorescence in FFPE pancreatic sections. Representative images of all cases showing expression of insulin (red), glucagon (blue) and ACE2 (MAB933, green), in human pancreatic FFPE sections obtained from seven different adult non-diabetic multiorgan donors.

A

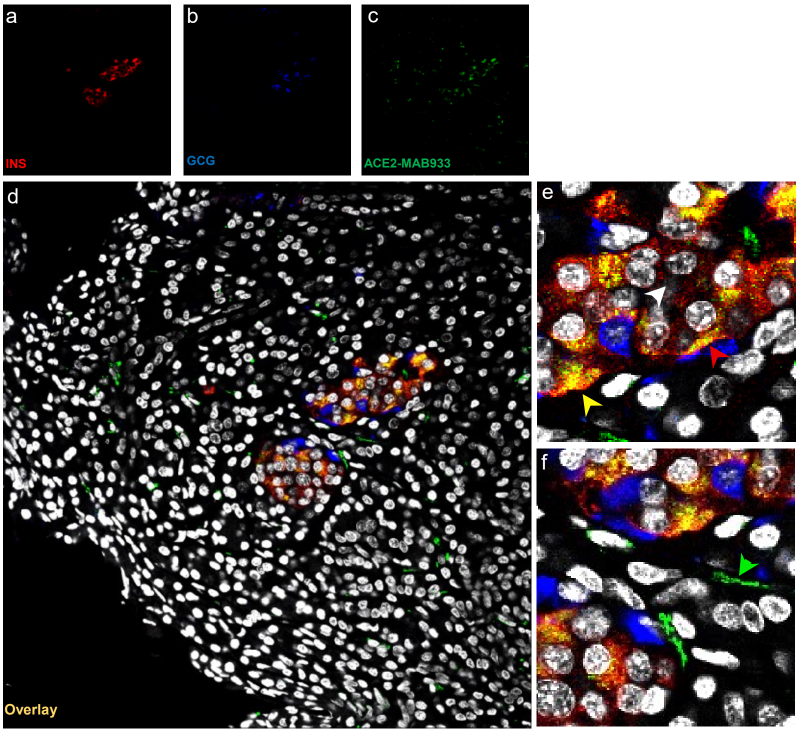

B

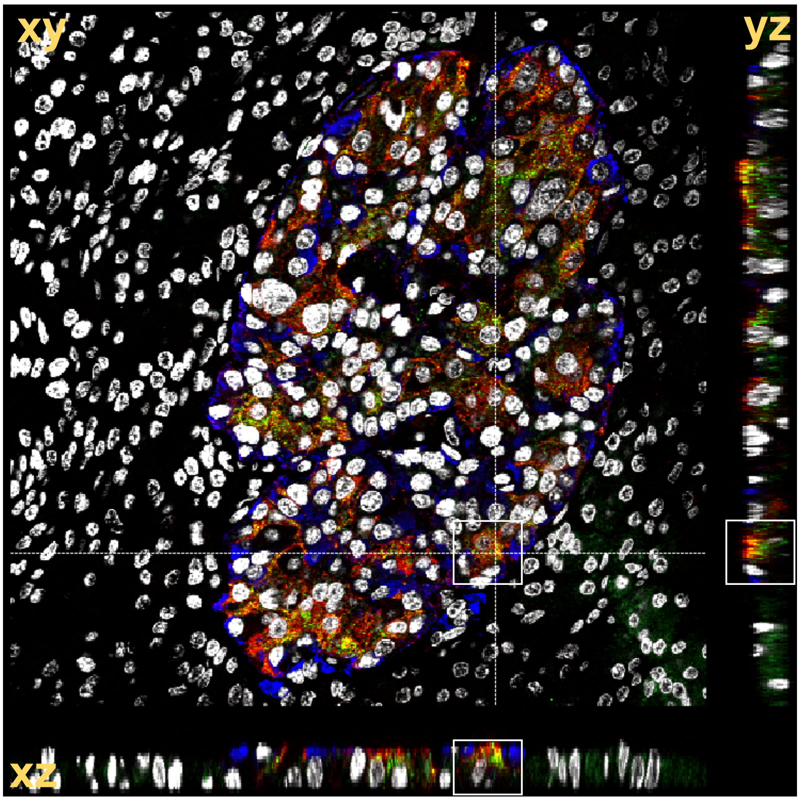

Figure S5. (**A**) Triple immunofluorescence staining and image analysis of FFPE human pancreatic section (case #210518) stained for insulin (red, panel-a), glucagon (blue, panel-b) and ACE2 MAB933 (green, panel-c), alongside with overlay (panel-d) and zoom overlays (panel-e and -f). White-arrow: ACE2-negative β-cell; yellow-arrow: granular/cytoplasmatic ACE2 signal in a β-cell;

red-arrow: ACE2-positivity prevalently associated to plasma membrane in a β-cell; green-arrow: plasma membrane localization of ACE2 in microvasculature-associated pericyte.

(B) Z-stack 3D analysis of a pancreatic islet stained for ACE2 (MAB933), insulin and glucagon. Representative image of a human pancreatic islet of case 210518 analysed performing triple immunofluorescence staining for ACE2 (green), insulin (red), glucagon (blue) and DAPI (white). Acquisition of 40 different focal planes allowed a XYZ 3D reconstruction and z-sectioning (xz and yz). White rectangles highlight an example of the partial colocalization of ACE2 and insulin signals within β-cells, highlighting the different subcellular compartmentalization (cytoplasmatic/granular and plasma membrane) of ACE2.

A

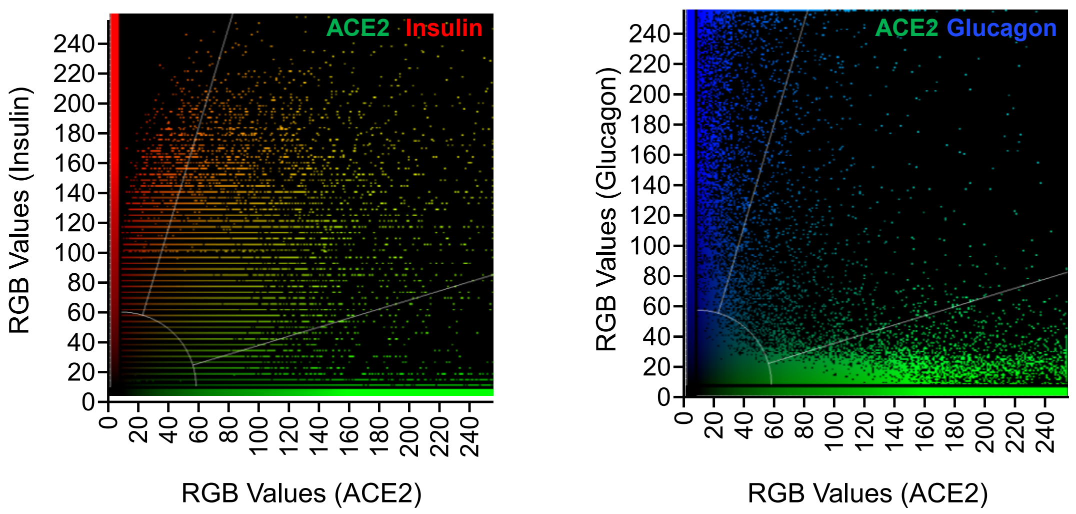

B

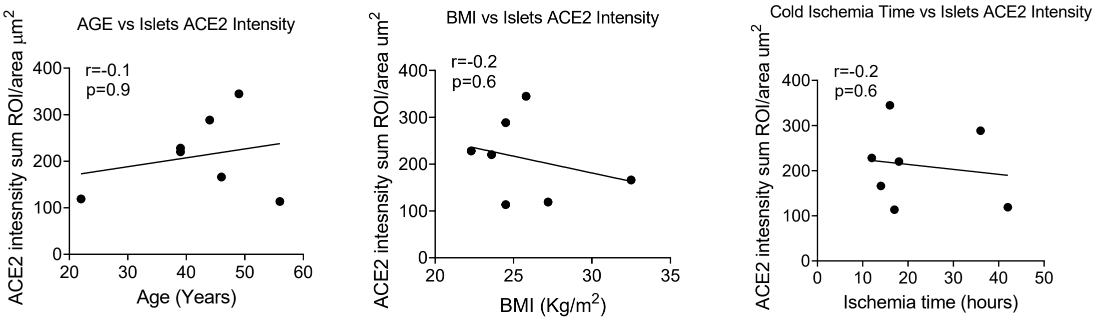

Figure S6. (A) Colocalization plots of ACE2-insulin and ACE2-glucagon in a human pancreatic islet of case 210518. (**B**) Correlation analysis among ACE2 pancreatic islets staining intensity of all cases analyzed (see Figure 4C) and Age (years), BMI (kg/m^2) and cold ischemia time (hours). Statistics performed using Spearman R correlation test.

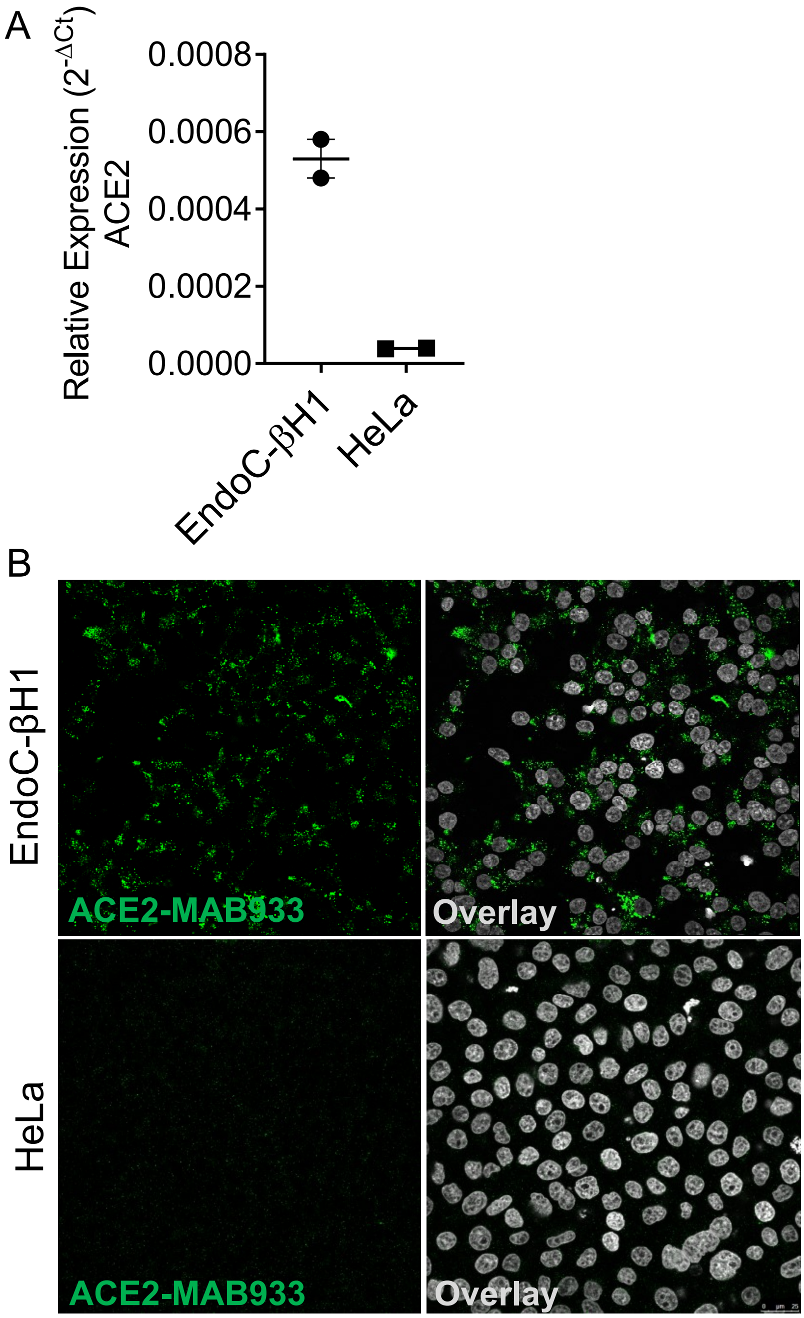

Figure S7. (A) Orthogonal validation of ACE2 R&D MAB933 antibody in cultured cell lines. ACE2 mRNA expression using qRT-PCR in EndoC-βH1 and HeLa cells. Cycle Threshold values of ACE2 were normalized using β-Actin and GAPDH values. Final values are reported as normalized 2^{-dCt} relative values. (B) Immunofluorescence analysis of ACE2 (R&D, MAB933) in EndoC-βH1 and HeLa cells stained using the same protocol.

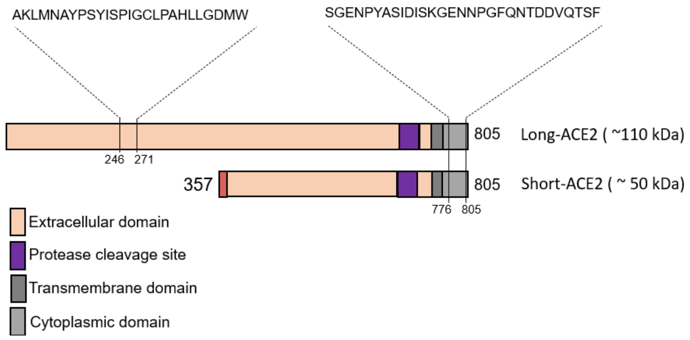


```
# Aligned_sequences: 2
# 1: EMBOSS_001
# 2: EMBOSS_001
# Matrix: EBLOSUM62
# Gap_penalty: 10.0
# Extend_penalty: 0.5
#
# Length: 805
# Identity:     452/805 (56.1%)
# Similarity:   452/805 (56.1%)
# Gaps:         346/805 (43.0%)
# Score: 2387.0
#

EMBOSS_001         1 MSSSSWLLLSLVAVTAAQSTIEEQAKTFLDKFNHEAEDLFYQSSLASWNY     50

EMBOSS_001         1 --------------------------------------------------      0

EMBOSS_001        51 NTNITEENVQNMNNAGDKWSAFLKEQSTLAQMYPLQEIQNLTVKLQLQAL    100

EMBOSS_001         1 --------------------------------------------------      0

EMBOSS_001       101 QQNGSSVLSEDKSKRLNTILNTMSTIYSTGKVCNPDNPQECLLLEPGLNE    150

EMBOSS_001         1 --------------------------------------------------      0

EMBOSS_001       151 IMANSLDYNERLWAWESWRSEVGKQLRPLYEEYVVLKNEMARANHYEDYG    200

EMBOSS_001         1 --------------------------------------------------      0

EMBOSS_001       201 DYWRGDYEVNGVDGYDYSRGQLIEDVEHTFEEIKPLYEHLHAYVRAKLMN    250

EMBOSS_001         1 --------------------------------------------------      0

EMBOSS_001       251 AYPSYISPIGCLPAHLLGDMWGRFWTNLYSLTVPFGQKPNIDVTDAMVDQ    300

EMBOSS_001         1 --------------------------------------------------      0

EMBOSS_001       301 AWDAQRIFKEAEKFFVSVGLPNMTQGFWENSMLTDPGNVQKAVCHPTAWD    350
                                                                .....||
EMBOSS_001         1 -------------------------------------------MREAGWD      7

EMBOSS_001       351 LGKGDFRILMCTKVTMDDFLTAHHEMGHIQYDMAYAAQPFLLRNGANEGF    400
                     .|.   ||||||||||||||||||||||||||||||||||||||||||||
EMBOSS_001         8 KGG---RILMCTKVTMDDFLTAHHEMGHIQYDMAYAAQPFLLRNGANEGF     54

EMBOSS_001       401 HEAVGEIMSLSAATPKHLKSIGLLSPDFQEDNETEINFLLKQALTIVGTL    450
                     ||||||||||||||||||||||||||||||||||||||||||||||||||
EMBOSS_001        55 HEAVGEIMSLSAATPKHLKSIGLLSPDFQEDNETEINFLLKQALTIVGTL    104

EMBOSS_001       451 PFTYMLEKWRWMVFKGEIPKDQWMKKWWEMKREIVGVVEPVPHDETYCDP    500
                     ||||||||||||||||||||||||||||||||||||||||||||||||||
EMBOSS_001       105 PFTYMLEKWRWMVFKGEIPKDQWMKKWWEMKREIVGVVEPVPHDETYCDP    154

EMBOSS_001       501 ASLFHVSNDYSFIRYYTRTLYQFQFQEALCQAAKHEGPLHKCDISNSTEA    550
                     ||||||||||||||||||||||||||||||||||||||||||||||||||
EMBOSS_001       155 ASLFHVSNDYSFIRYYTRTLYQFQFQEALCQAAKHEGPLHKCDISNSTEA    204

EMBOSS_001       551 GQKLFNMLRLGKSEPWTLALENVVGAKNMNVRPLLNYFEPLFTWLKDQNK    600
                     ||||||||||||||||||||||||||||||||||||||||||||||||||
EMBOSS_001       205 GQKLFNMLRLGKSEPWTLALENVVGAKNMNVRPLLNYFEPLFTWLKDQNK    254

EMBOSS_001       601 NSFVGWSTDWSPYADQSIKVRISLKSALGDKAYEWNDNEMYLFRSSVAYA    650
                     ||||||||||||||||||||||||||||||||||||||||||||||||||
EMBOSS_001       255 NSFVGWSTDWSPYADQSIKVRISLKSALGDKAYEWNDNEMYLFRSSVAYA    304

EMBOSS_001       651 MRQYFLKVKNQMILFGEEDVRVANLKPRISFNFFVTAPKNVSDIIPRTEV    700
                     ||||||||||||||||||||||||||||||||||||||||||||||||||
EMBOSS_001       305 MRQYFLKVKNQMILFGEEDVRVANLKPRISFNFFVTAPKNVSDIIPRTEV    354

EMBOSS_001       701 EKAIRMSRSRINDAFRLNDNSLEFLGIQPTLGPPNQPPVSIWLIVFGVVM    750
                     ||||||||||||||||||||||||||||||||||||||||||||||||||
EMBOSS_001       355 EKAIRMSRSRINDAFRLNDNSLEFLGIQPTLGPPNQPPVSIWLIVFGVVM    404

EMBOSS_001       751 GVIVVGIVILIFTGIRDRKKKNKARSGENPYASIDISKGENNPGFQNTDD    800
                     ||||||||||||||||||||||||||||||||||||||||||||||||||
EMBOSS_001       405 GVIVVGIVILIFTGIRDRKKKNKARSGENPYASIDISKGENNPGFQNTDD    454

EMBOSS_001       801 VQTSF    805
                     |||||
EMBOSS 001       455 VQTSF    459
```

Figure S8. ACE2 Targeted Mass Spectrometry-Shotgun Proteomic Analysis of EndoC-βH1. (A) Representative scheme of ACE2 isoforms (short-ACE2 and long-ACE2) and consistent peptides identified in EndoC-βH1 using shotgun proteomics in n=2 independent experiments. Below**,** alignment of ACE2 isoforms protein FASTA sequences performed using EMBOSS. Red sequence: short-ACE2; black sequence: long-ACE2; yellow highlighted sequences: shotgun proteomics identified peptides**.**

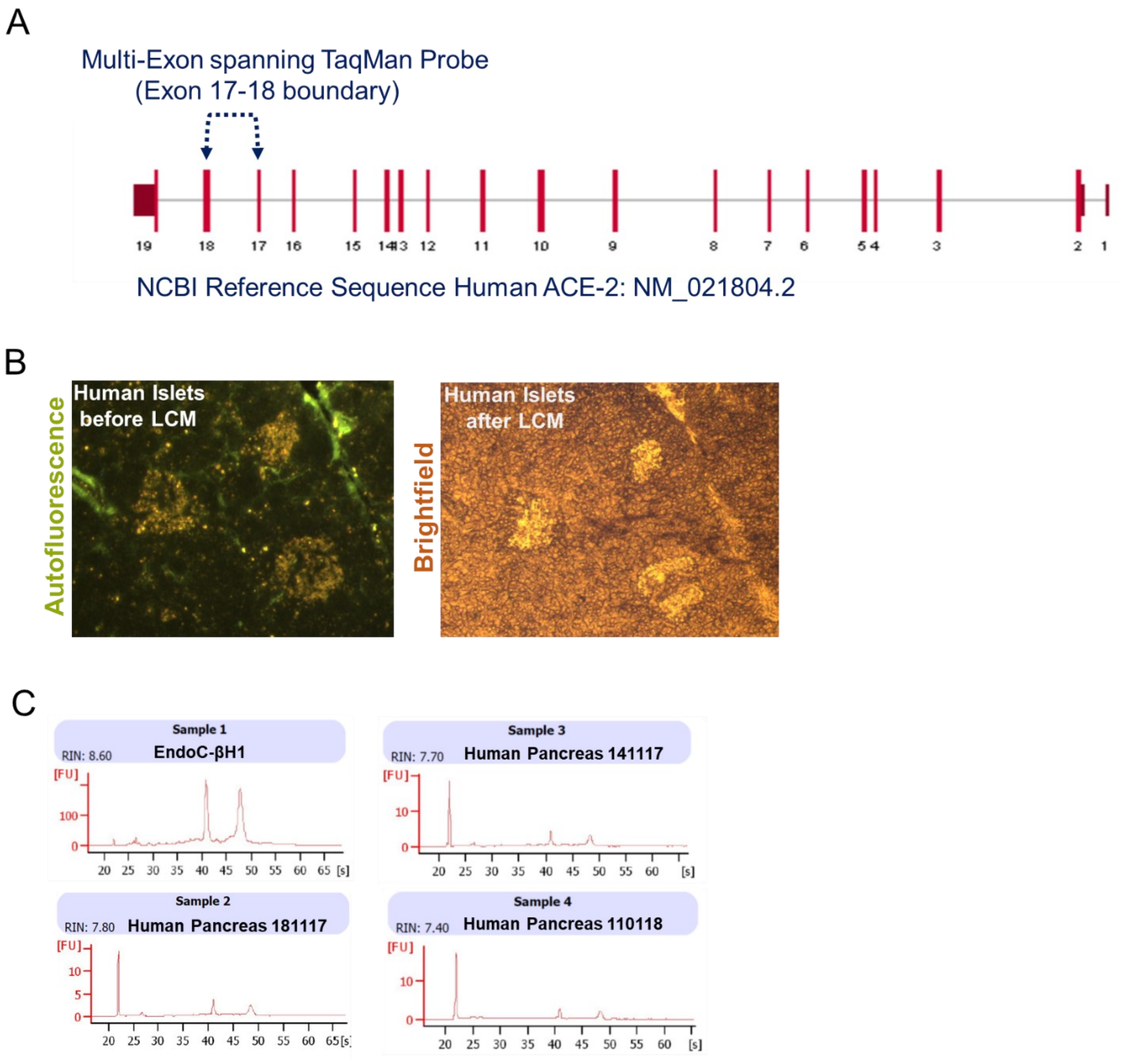

Figure S9. ACE2 mRNA expression. (A) ACE2 gene scheme reporting exons of full length transcript (Ref Seq: NM_021804.2) and showing the location of the TaqMan Assay (Hs01085333_m1, assay location 2332nt, exon 17-18, amplicon length:141nt) used to detect ACE2 mRNA expression. (**B**) Human pancreatic islets autofluorescence and brightfield images in a human pancreatic frozen section, before and after laser capture microdissection procedure. (C) Electropherogram of total RNA extracted from EndoC-βH1 cells and from LCM-islets from human pancreatic sections obtained from five non-diabetic multiorgan donors. Representative RNA Integrity Number (RIN) is reported for each RNA sample analyzed.

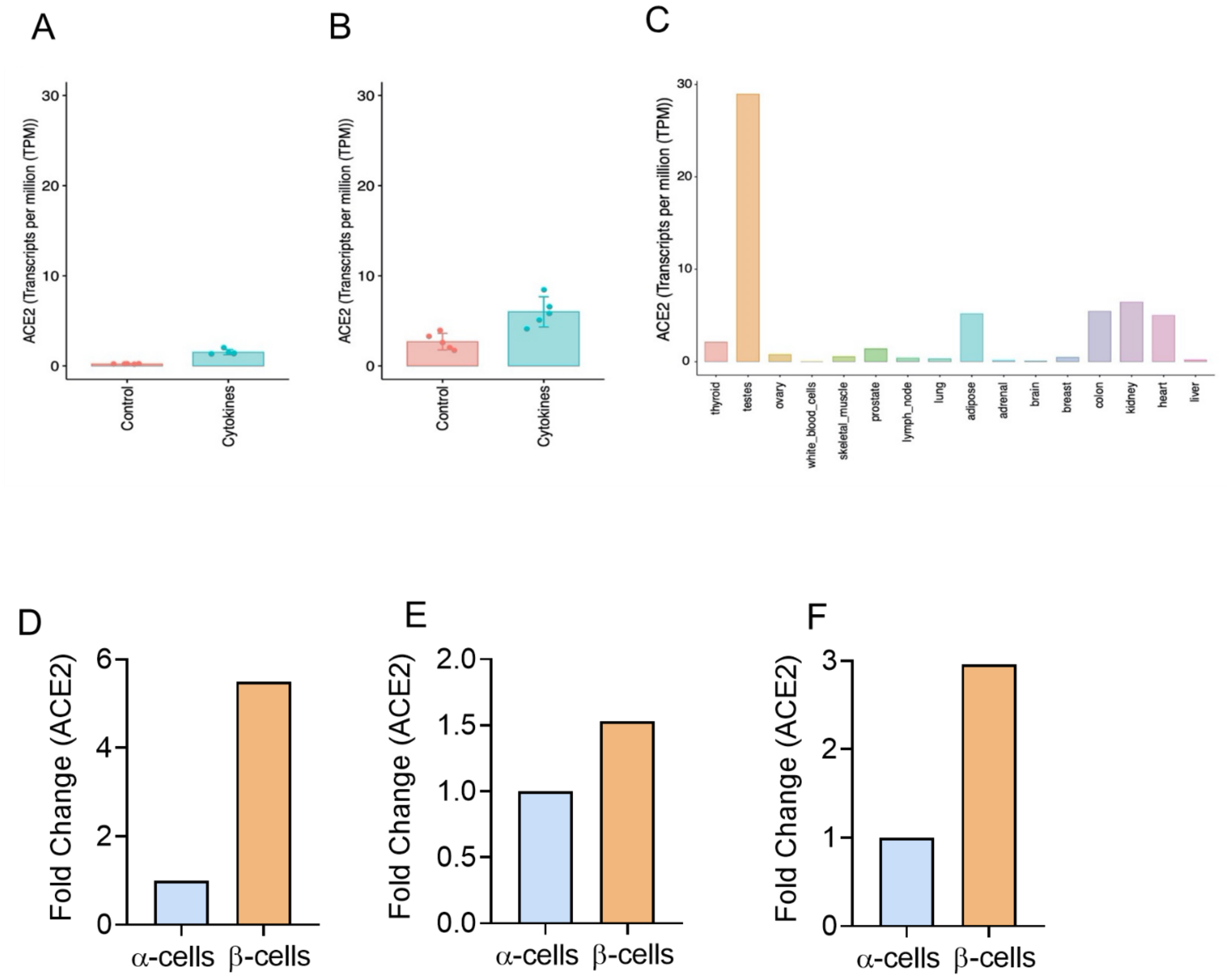

Figure S10. Expression of ACE2 mRNA expression in different conditions and tissues. The expression of ACE2 was evaluated in EndoC-βH1 cells (**A**) and pancreatic human islets (**B**) exposed or not to proinflammatory cytokines (IL1β + IFNγ) for 48h and in 16 different tissues from the Illumina Body Map 2.0 (GEO: GSE30554)) (**C**) that were sequenced at a similar deep coverage (coverage > 150 x 106 reads / per sample). Values are represented as transcripts per million (TPM). All the samples were processed using the same RNA-seq pipeline as described in Methods.

(D-E) ACE2 expression in β-cells and in α-cells, obtained from three different RNA-seq datasets. Data were extrapolated from supplementary files of normalized and analyzed datasets obtained from three different published studies which compared β- and α-cells transcriptomes. In (**D**) Dorrell et al 2011, in (**E**) Bramswig et al 2013, and in (**F**) Blodgett et al 2015 (Blodgett et al., 2015; Bramswig et al., 2013; Dorrell et al., 2011).

A

ACTGCTGTCCCAGGCTCCTTGTTCATTTATTCTTAGCTTTCAGATTTTATATCTTGCTTT
CTCCATTTCAGTTTGATCAAGTTAATCGTATTTTCTGCCTCAGCGAATTGAATTTGGACT
A**CTCCCATTTTTTCCACTAAACC**CACGTGTTTCATTTAGGAACTTACCTGTGTAGAAATT
STAT1

TATTTCCGCTTTTA**TTACAGTAA**CATTTCAACCTTTTCTTCAGATACTCGTGTGCTGTCC
STAT

TCTTTCTTGCCTGTTTTTTCTCCTATTGTGTGCAAGGAGTGAGT**GGATCTAGGTTTCCTG**
STAT1
STAT3
STAT5
STAT

GAATGTGGGAGGAGCTTTTCTGAAATTTCTGCACTGATTTTTTTTTTTTTACCCTCATCTC

ACTTTCATTTAAAATTCTTTATTATTATTACTTATATTTGTGAGCTGCTTTATTTTTTTA
AAAGGAGAGTATATTTTGATTCACATCAAGGGAAGTAAGAGTCCACCTCTTTTTGATGCA
GTGTAACTAAATCTGTCATCTTCACTGAGGCACAAGGCCCTCTGTCACATTTTATCTATG
CCTCCACTATTGCTTTTAGACTGTTGACTACAAGTTAGTTAAGAGCCTATACTCTAGAAT
CAGAATACATAGATCCATGTTCTGATTCCATCATTTGTTAGCTGAGTGAGATATAGCAAT
TTACTTAAACTTAGTTTCATAATTCATAAAATGGTGATAAAATATTTGCCTATAGGTTTC
TTGTGATGCTCAAATGAGATATTATAACACAAAGCACTTTGAATAGTTACTTATAATTGT
TTTTTTCCTATTCTACAATAATATACACAACTTTGGAATAAGGAAAAGCAGTGGACATTT
TTTTAAAGGCTTGATTATTGCAATGTCACCTGAACCTGGAAGACTTGTTTTTCTGGGTGA
AGAAATATTTTCTCTGTGTCAGAGTTTCACAATCATCGTCAGGTAGGCCCTTGAACCCTG
CCATTTAAAGTGCTCCTCTCTTTGATCTGTGGCACTCATA

Rank	P-value	Matrix_ID	Matrix_name
1	0,0009	M01478	V$CPHX_01
2	0,0043	M00412	V$AREB6_01
3	0,0063	M00250	V$GFI1_01
4	0,0084	M01058	V$GFI1B_01
5	0,0100	M01232	V$SATB1_01
**6**	**0,0117**	**M00457**	**V$STAT5A_01**
**7**	**0,0118**	**M01260**	**V$STAT1_05**
8	0,0119	M00119	V$MAX_01
**9**	**0,0124**	**M00459**	**V$STAT5B_01**
10	0,0129	M00347	V$GATA1_06
11	0,0129	M01004	V$HELIOSA_02
12	0,0157	M00711	V$ZTA_Q2
13	0,0160	M00317	V$LDSPOLYA_B
14	0,0180	M01446	V$BARHL2_01
15	0,0190	M01022	V$LEF1_Q2_01
16	0,0191	M01116	V$CLOCKBMAL_Q6
17	0,0196	M00615	V$MYCMAX_03
18	0,0198	M01425	V$HNF1B_01
19	0,0201	M01014	V$SOX_Q6
20	0,0214	M00632	V$GATA4_Q3
21	0,0216	M00318	V$LPOLYA_B
22	0,0217	M00802	V$PIT1_Q6
23	0,0231	M01665	V$IRF8_Q6
**24**	**0,0251**	**M00223**	**V$STAT_01**
25	0,0251	M01352	V$NKX29_01
26	0,0311	M01124	V$OCT4_02
27	0,0323	M00302	V$NFAT_Q6
28	0,0335	M01185	V$BCL6_02
29	0,0346	M00033	V$P300_01
30	0,0367	M00799	V$MYC_Q2
31	0,0376	M00055	V$NMYC_01
32	0,0384	M01009	V$HES1_Q2
33	0,0391	M00451	V$NKX3A_01
34	0,0399	M01383	V$NKX3A_02
35	0,0405	M00671	V$TCF4_Q5
36	0,0407	M00320	V$MTATA_B
37	0,0408	M00118	V$MYCMAX_01
38	0,0423	M01464	V$HOXA10_01
39	0,0430	M00123	V$MYCMAX_02
40	0,0441	M00472	V$FOXO4_01
41	0,0458	M01342	V$CDP_03
42	0,0459	M00059	V$YY1_01
**43**	**0,0460**	**M01595**	**V$STAT3_03**
44	0,0469	M01457	V$NKX23_01

B

ACTGCTGTCCCAGGCTCCTTGTTCATTTATTCTTAGCTTTCAGATTTTATATCTTGCTTT
CTCCATTTCAGTTTGATCAAGTTAATCGTATTTTCTGCCTCAGCGAATTGAATTTGGACT
ACTCCCAT**TTTTTCCACTAAACC**CACGTGTTTCATTTAGGAACTTACCTGTGTAGAAATT
STAT1

TATTTCCGCTTTTATTACAGTAACATTTCAACCTTTTCTTCAGATACTCGTGTGCTGTCC

TCTTTCTTGCCTGTTTTTTCTCCTATTGTGTGCAAGGAGTGAGTGGATCTA**GGTTTCCTG**
STAT1
STAT3

GAATGTGGGAGGAGCTTTTCTGAAATTTCTGCACTGATTTTTTTTTTTTTACCCTCATCTC

ACTTTCATTTAAAATTCTTTATTATTATTACTTATATTTGTGAGCTGCTTTATTTTTTTA
AAAGGAGAGTATATTTTGATTCACATCAAGGGAAGTAAGAGTCCACCTCTTTTTGATGCA
GTGTAACTAAATCTGTCATCTTCACTGAGGCACAAGGCCCTCTGTCACATTTTATCTATG
CCTCCACTATTGCTTTTAGACTGTTGACTACAAGTTAGTTAAGAGCCTATACTCTAGAAT
CAGAATACATAGATCCATGTTCTGATTCCATCATTTGTTAGCTGAGTGAGATATAGCAAT
TTACTTAAACTTAGTTTCATAATTCATAAAATGGTGATAAAATATTTGCCTATAGGTTTC
TTGTGATGCTCAAATGAGATATTATAACACAAAGCACTTTGAATAGTTACTTATAATTGT
TTTTTTCCTATTCTACAATAATATACACAACTTTGGAATAAGGAAAAGCAGTGGACATTT
TTTTAAAGGCTTGATTATTGCAATGTCACCTGAACCTGGAAGACTTGTTTTTCTGGGTGA
AGAAATATTTTCTCTGTGTCAGAGTTTCACAATCATCGTCAGGTAGGCCCTTGAACCCTG
CCATTTAAAGTGCTCCTCTCTTTGATCTGTGGCACTCATA

Rank	P-value	Matrix_ID	Matrix_name
**1**	**0,0080**	**MA0144.1**	**STAT3**
2	0,0134	MA0058.1	MAX
**3**	**0,0224**	**MA0137.2**	**STAT1**
4	0,0278	MA0093.1	USF1
5	0,0317	MA0258.1	ESR2
6	0,0384	MA0004.1	Arnt
7	0,0489	MA0150.1	NFE2L2

Figure S11. ACE2 promoter Transcription factor binding motifs analysis. TRAP analysis of transcription factors binding motifs in 1000 bp upstream sequence from ACE2 TSS (ACE2 proximal promoter). Analysis of TFs binding motifs using TRANSFAC database (**A**) and JASPAR database (**B**), alongside with related tables reporting ranking, p-values, TF matrix ID and TF binding motifs

official name. STATs binding motifs are highlighted in different colours within ACE2 promoter sequences and reported in bold in the related table.

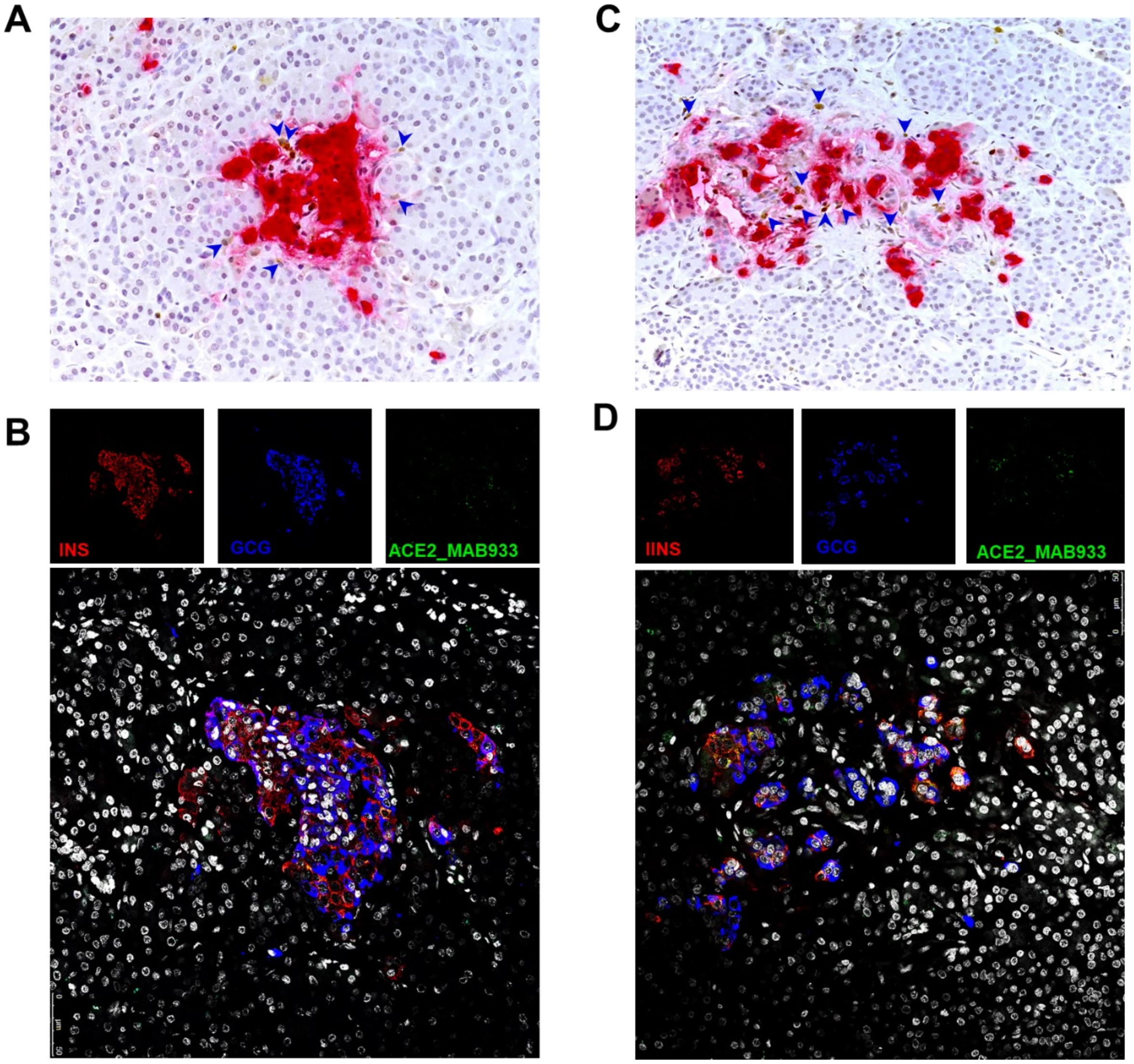

Figure S12. Triple immunofluorescence analysis of insulin (red), glucagon (blue) and ACE2 MAB933 (green) in a FFPE section of a longstanding T1D donor. Two infiltrated islets (A-B and C-D) were identified based on IHC insulin (red)-CD45 (brown) staining in a previous serial sections of the same T1D case.

Table S1. Characteristics of non-diabetic multiorgan donors. Main descriptive characteristics are reported alongside with tissue type obtained. Human islets (HI) purity values (only for pancreatic islets enzymatic isolation) and type of analysis performed, are reported as well.

Case/ Sample ID	Gender	Age (years)	BMI (Kg/m2)	AutoAb (ELISA)	HiRes HLA	Ischemia Time (hours)	Cause of Death	Tissue type	HI Purity (Dithizone Stain- %)	HI Purity (insulin ICC-%)	Used for
141117 CTR	M	49	25.8	GADA neg, IA-2A neg, ZnT8A neg	HLA:A*03,68; B*35,47; C*04,06; DRB1*03,08; DQB1*02,04	16h	Cardiovascular disease	FFPEand fresh-frozen pancreatic tissue	--	--	IHC/LCM
181117 CTR	M	39	23.6	GADA neg, IA-2A neg ZnT8A neg	HLA:A*03,33; B*14, B*14 SD B65; C*08; DRB1*01, DQB1*05	18h	Trauma	FFPE and fresh-frozen pancreatic tissue	--	--	IHC/LCM
110118 CTR	F	46	32.5	GADA neg IA-2A neg, ZnT8A neg	HLA:A*24; B*15,18; C*7; DRB1*04,11; DQB1*3	14h	Cardiovascular disease	FFPE and fresh-frozen pancreatic tissue	--	--	IHC/LCM
300318 CTR	F	56	24.5	GADA neg, IA-2A neg, ZnT8A neg	HLA:A*2; B*07,50; C*06,07; DRB1*03,15; DQB1*02,06	17h	Trauma	FFPE and fresh-frozen pancreatic tissue	--	--	IHC
210518 CTR	M	44	24.5	GADA neg, IA-2A neg, ZnT8A neg	HLA:A*24,34; B*15,55; C*03,07; DRB1*11,13; DQB1*3	36	Cardiovascular disease	FFPE and fresh-frozen pancreatic tissue	--	--	IHC
161118 CTR	F	39	22.3	GADA neg, IA-2A neg, ZnT8A neg	HLA:A LR*03,68; B*07,51; C*07,14; DRB1*01,15; DQB1*05,06	12h	Cardiovascular disease	FFPE and fresh-frozen pancreatic tissue	--	--	IHC/LCM
110119 CTR	M	22	27.2	GADA neg, IA-2A neg, ZnT8A neg	HLA:A*01,02; B*08,18; C*07,07; DRB1*03,11; DQB1*02,03	42h	Trauma	FFPE and fresh-frozen pancreatic tissue	--	--	IHC/LCM
060217	F	39<br>T1D duration :21	24,5	GADA pos, IA-2A neg, ZnT8A neg	HLA:A*01,02; B*08,B*50; C*07, C*12; DRB1*03, DQRB1*17, DQB1*02	43h	Trauma	FFPE and fresh-frozen pancreatic tissue	--	--	IHC
Islet prep 1	F	23	22.5	n/a	n/a	--	Cardiac arrest	Enzymatic-isolated pancreatic islets	--	46	RNA-seq (IL-1β + IFNγ treatment experiments)
Islet prep 2	M	31	27.8	n/a	n/a	--	Cerebral hemorrhage	Enzymatic-isolated pancreatic islets	--	66	RNA-seq (IL-1β + IFNγ treatment experiments)
Islet prep 3	M	77	24.5	n/a	n/a	--	Cerebral hemorrhage	Enzymatic-isolated pancreatic islets	--	59	RNA-seq (IL-1β + IFNγ treatment experiments)
Islet prep 4	F	64	29.4	n/a	n/a	--	Cerebral hemorrhage	Enzymatic-isolated pancreatic islets	--	47	RNA-seq (IL-1β + IFNγ treatment experiments)
Islet prep 5	F	58	21.3	n/a	n/a	--	Cerebral hemorrhage	Enzymatic-isolated pancreatic islets	--	67	RNA-seq (IL-1β + IFNγ treatment experiments)

Islet prep 6	M	67	25.7	n/a	n/a	--	Trauma	Enzymatic-isolated pancreatic islets	--	48	RNA-seq (IFNα treatment experiments)
Islet prep 7	F	87	23.8	n/a	n/a	--	Cerebral hemorrhage	Enzymatic-isolated pancreatic islets	--	60	RNA-seq (IFNα treatment experiments)
Islet prep 8	F	67	24.6	n/a	n/a	--	Cerebral hemorrhage	Enzymatic-isolated pancreatic islets	--	44	RNA-seq (IFNα treatment experiments)
Islet prep 9	F	83	37.1	n/a	n/a	--	CVD	Enzymatic-isolated pancreatic islets	--	51	RNA-seq (IFNα treatment experiments)
Islet prep 10	F	84	24.5	n/a	n/a	--	CVD	Enzymatic-isolated pancreatic islets	--	58	RNA-seq (IFNα treatment experiments)
Islet prep 11	F	40	22.5	n/a	n/a	--	Stroke	Enzymatic-isolated pancreatic islets	--	59	RNA-seq (IFNα treatment experiments)
Hi#2	M	52	34.2	n/a	n/a	--	n/a	Enzymatic-isolated pancreatic islets	≥70%		qRT-PCR ACE2 mRNA
Hi#3	M	50	27.4	n/a	n/a	--	n/a	Enzymatic-isolated pancreatic islets	≥70%		qRT-PCR ACE2 mRNA
Hi#4	M	55	28	n/a	n/a	--	n/a	Enzymatic-isolated pancreatic islets	≥70%		qRT-PCR ACE2 mRNA
Hi#8	M	38	23.1	n/a	n/a	--	Cardiovascular disease	Enzymatic-isolated pancreatic islets	90%		qRT-PCR ACE2 mRNA

Table S2. ACE2-Insulin and ACE2-Glucagon colocalization rate (reported as percentage values) in human pancreatic islets of EUnPOD non-diabetic cases. Two different FFPE pancreas blocks per case were analysed; results for each block are reported separately in the table below.

Case ID	Pancreas Block ID	ACE2-INS (Colocalization Rate- %)	ACE2-GCG (Colocalization Rate- %)
110118	Body 01A	47.5%	8.3%
110118	Head 02A	59.3%	12.2%
110119	Body 01A	63.2%	3.1%
110119	Tail 01A	59.3%	2.3%
141117	Body 01B	83.6%	6.6%
141117	Tail 03B	55.0%	5.6%
161118	Body 01A	73.9%	6.8%
161118	Tail 01A	81.9%	12.6%
181117	Head 02B	53.6%	7.0%
181117	Tail 01A	62.2%	9.2%
210518	Body 01B	39.5%	7.5%
210518	Head 02A	35.6%	11.3%
300318	Body 01A	61.8%	4.1%
300318	Tail 01A	39.0%	1.5%

Table S3. Expression of ACE2 mRNA (ENST00000252519.8) in RNA-seq dataset of five different samples of EndoC-βH1, and five different Human Pancreatic islets preparations exposed or not to IL-1β + IFNγ (48h), and five different samples of EndoC-βH1 and six of Human Pancreatic islets exposed or not to IFNα (18h).

**IL-1β + IFNγ (48h)**				
	Not treated	Treated (IL-1β + IFNγ)	Log2 Fold Change (Cyt vs. nt)	pValue Adj
EndoC-βH1	0.061	1.05	4.19	3.02E-35
Human Pancreatic islets	2.20	5.76	1.51	7.902E-17
**IFNα (18h)**				
	Not treated	Treated (IFNα)	Log2 Fold Change (Cyt vs. nt)	pValue Adj
EndoC-βH1	0.057	3.15	5.92	9.35E-42
Human Pancreatic islets	3.17	16.30	2.39	8.45E-26

Reagents Table. Table reporting reagents or resources used in this study.

REAGENT or RESOURCE	SOURCE	IDENTIFIER
Antibodies and reagent for IHC/IFA		
Monoclonal Mouse anti-Human ACE2	R&D System	MAB933, RRID:AB_2223153
Monoclonal Rabbit anti-Human ACE2	Abcam	Ab108252 RRID:AB_10864415
Polyclonal Rabbit anti-Human ACE2	Abcam	Ab15348 RRID:AB_301861
Polyclonal Rabbit anti-Human CD31	Abcam	Ab28364 RRID:AB_726362
Polyclonal Rabbit Anti-Human Glucagon	Agilent/Dako	A0565 RRID:AB_10013726
Monoclonal Mouse Anti-Human Glucagon	R&D System	MAB1249 RRID:AB_2107340
FLEX Polyclonal Guinea Pig Anti-Insulin, Ready-to-Use	Agilent/Dako	IR002 RRID:AB_2800361
Polyclonal Rabbit Anti-Mouse/HRP	Agilent/Dako	P0260 RRID:AB_2636929
Polyclonal Goat Anti-Rabbit/HRP	Jackson ImmunoResearch Labs	111-036-003 RRID:AB_2337942
Goat Anti-Mouse IgG (H+L) Highly Cross-adsorbed Antibody, Alexa Fluor 488	Thermo Fisher Scientific	A-11029 RRID:AB_2534088
Goat anti-Rabbit IgG (H+L) Highly Cross-Adsorbed Antibody, Alexa Fluor 488	Thermo Fisher Scientific	A-11034 RRID:AB_2576217
Goat anti-Guinea Pig IgG (H+L) Highly Cross-Adsorbed Antibody, Alexa Fluor 555	Thermo Fisher Scientific	A-21435 RRID:AB_2535856
Goat anti-Guinea Pig IgG (H+L) Highly Cross-Adsorbed Antibody, Alexa Fluor 594	Thermo Fisher Scientific	A-11076 RRID:AB_2534120
Goat anti-Rabbit IgG (H+L) Highly Cross-Adsorbed Antibody, Alexa Fluor 594	Thermo Fisher Scientific	A-11037 RRID:AB_2534095
Goat anti-Rabbit IgG (H+L) Highly Cross-Adsorbed Antibody, Alexa Fluor 647	Thermo Fisher Scientific	A-21245 RRID:AB_2535813
Goat anti-Mouse IgG (H+L) Highly Cross-Adsorbed Antibody, Alexa Fluor 647	Thermo Fisher Scientific	A-21236 RRID:AB_2535805
Biological Samples		
Human pancreatic islets	Tebu Bio/ University of Pisa	N/A
Human pancreatic tissues	EUnPOD INNODIA	N/A
Human lung tissues	University of Siena	N/A
Chemicals		
Absolute Ethanol	VWR	1.009.862.500
Xilene	Merck	28975325
Hematoxylin	Sigma Aldrich	MHS31
β-Mercaptoethanol	Sigma Aldrich	M7522
LCM caps	Thermo Fisher Scientific	LCM0214
DAPI	Sigma Aldrich	D8517
Citrate buffer:<br>0.1 M Citric Acid Monohydrate<br>0.1M Sodium Citrate Tribasic Dihydrate	 Sigma Aldrich<br>Sigma Aldrich	 C1909<br>S4641
Critical Commercial Assays		
PicoPure RNA isolation kit Arcturus	Thermo Fisher Scientific	kit0204
Agilent 2100 Bioanalyzer technology with RNA Pico chips	Agilent Technologies	5067-1513
SuperScript™ VILO™ cDNA Synthesis Kit	Thermo Fisher Scientific	11754050

SensiFast Probe Lo-ROX Kit	Aurogene s.r.l	BIO-84020
Direct-zol RNA Miniprep Kit	Zymo Research	R202
Taq Man Preamp Master mix	Thermo Fisher Scientific	4488593
RNase-Free DNase Set	Qiagen	79254
Deposited Data		
RNA-seq human islets and EndoC-βH1 exposed to IFNα	N/A	GSE133221
RNA-seq human islets exposed to IL-1 β + IFNγ	N/A	GSE108413
RNA-seq Endoc-βH1 exposed to IL-1 β + IFNγ	N/A	GSE137136
Experimental Models: Cell Lines		
EndoC-βH1 human β-cell line	UniverCell-Biosolutions (Toulouse-France)	N/A
Oligonucleotides		
Endogenous control human GAPDH	Thermo Fisher Scientific	4333764
Endogenous control human ACTB	Thermo Fisher Scientific	4333762
Taq Man gene expression assay ACE2	Thermo Fisher Scientific	Hs01085333
Software and Algorithms		
Expression Suite software 1.0.1	Thermo Fisher Scientific	N/A
Leica TCS SP5 confocal laser scanning microscope system	Leica Microsystems	N/A
Laser Advanced fluorescence	Leica Microsystems	N/A
Opera Phenix High Content Screening System	PerkinElmer	N/A
Harmony® Office	PerkinElmer	N/A
Graph Pad Prism 8	Prism	N/A
Qubit 3000 Fluorometer	Thermo Fisher Scientific	N/A
Other		
DMEM high-glucose	Sigma Aldrich	51441C
Penicillin/Streptomycin	Sigma Aldrich	P0781
ECM gel from Engelbreth-Holm swarm murine sarcoma	Sigma Aldrich	E1270
Fibronectin from bovine plasma	Sigma Aldrich	F1141
DMEM low-glucose	Sigma Aldrich	D6046
BSA fraction V	Sigma Aldrich	10775835001
L-Glutamine	Sigma Aldrich	G7513
Penicillin/Streptomycin	Sigma Aldrich	P0781
Nicotinamide	Sigma Aldrich	N0636
Transferrin	Sigma Aldrich	T8158
Sodium selenite	Sigma Aldrich	S5261
Sodium Palmitate	Sigma Aldrich	P9767-5G
IL-1β	R&D System	201-LB-005
TNFα	Sigma Aldrich	T7539
IFNγ	Roche	11040596001
CMRL culture medium	Thermo Fisher Scientific	11-530-037
Antibiotic/Antimicotic	Sigma Aldrich	A5955-100ML
Fetal Bovine Serum	Euroclone	EUS031153
EUKITT mounting medium	Bio Optica	S9-25-37
DAKO PAP pen	Agilent/Dako	S2002
PBS w/o Ca2+ Mg2+	Gibco	14040-091
Hydrogen peroxide	Sigma Aldrich	H1009

Bovine Serum Albumin	Sigma Aldrich	A1470-25G
Glycine	Thermo Fisher Scientific	100006373
DAB Novolink MAX DAB	Leica	RE7270-K
Vectashield	Vector Laboratories	H-1000
Rabbit serum	Sigma Aldrich	R9133
Goat serum	Sigma Aldrich	G9023
