# Supplementary material for: SARS-CoV-2 Receptor Angiotensin I-Converting Enzyme Type 2 (ACE2) Is Expressed in Human Pancreatic β-Cells and in the Human Pancreas Microvasculature
Source: Front Endocrinol (Lausanne). 2020 Nov 13;11:596898. doi: 10.3389/fendo.2020.596898 (PMC7691425; doi:10.3389/fendo.2020.596898)
Supplement: Supplementary file 2 [file DataSheet_2.pdf]

Sequence Coverage Map ACE2 in EndoC-βH1 (Exp1)

Minimum MS Signal = 40000
Protease = Trypsin

Proteins	Number of MS Peaks	MS Peak Area	Sequence Coverage	Abundance (mol)
1:ACE2	61	0.3%	39.1%	100.00%
Unidentified	54507	99.7%		

MS Signal Threshold = 5.4e+004

ACE2

1 MSSSSWLLLSLVAVTAAQSTIEEQAKTFLDKFNHEAEDLFYQSSLASWNY

51 NTNITEENVQNMNNAGDKWSAFLKEQSTLAQMYPLQEIQNLTVKLQLQAL

34.0

101 QQNGSSVLSEDKSKRLNTILNTMSTIYSTGKVCNPDNPQECLLLEPGLNE

34.0 | 71.9 | 25.1 | 21.5

151 IMANSLDYNERLWAWESWRSEVGKQLRPLYEEYVVLKNEMARANHYEDYG

33.0 | 3.2*

201 DYWRGDYEVNGVDGYDYSRGQLIEDVEHTFEEIKPLYEHLHAYVRAKLMN

44.7 | 52.0

251 AYPSYISPIGCLPAHLLGDMWGRFWTNLYSLTVPFGQKPNIDVTDAMVDQ

44.7 | 62.1 | 52.0 | 1.4

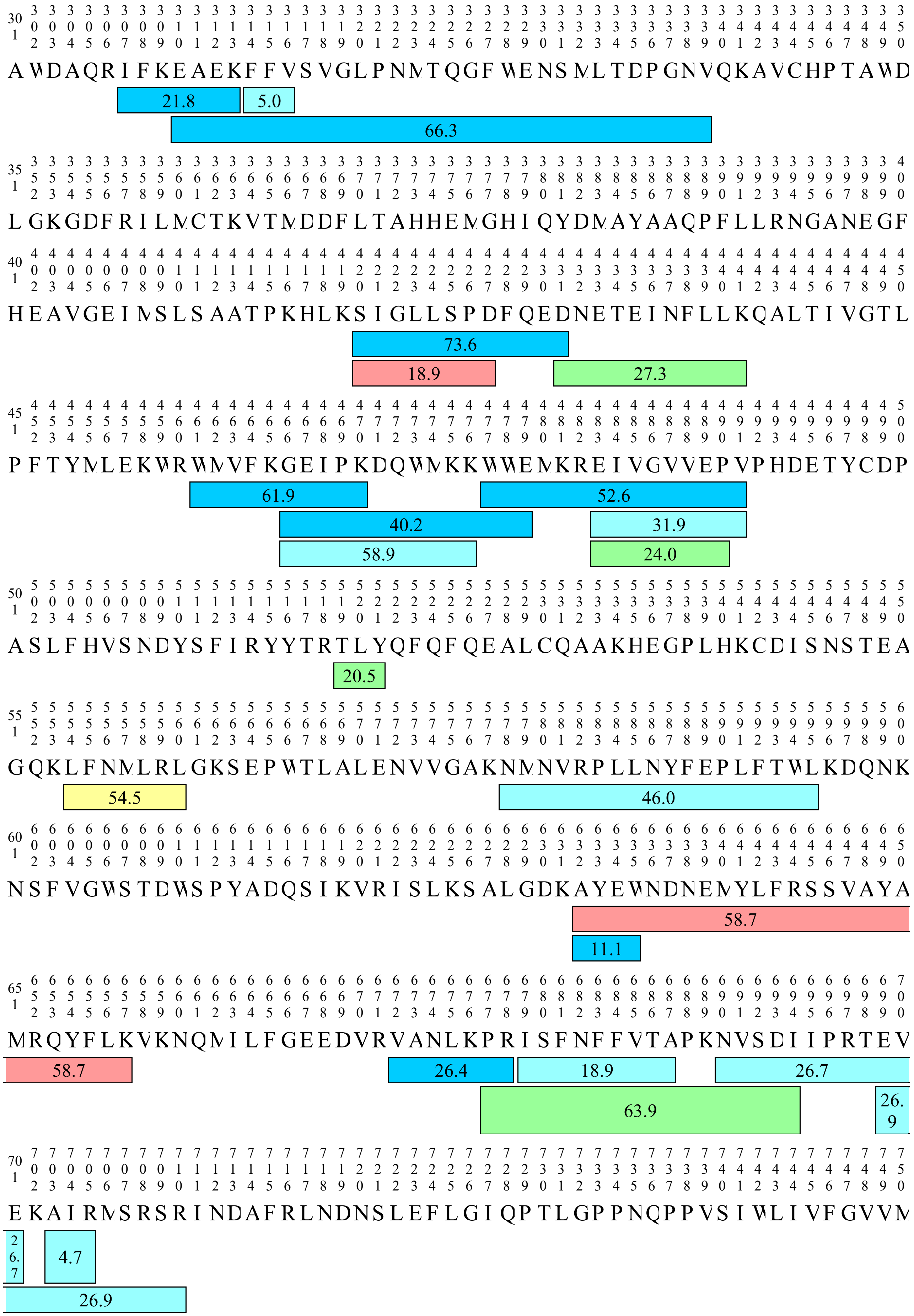
301
AWDAQRIFKEAEKFFVSVGLPNMTQGFWENSMLTDPGNVQKAVCHPTAWD
21.8
5.0
66.3
351
LGKGDFRILMCTKVTMDDFLTAHHEMGHIQYDMAYAAQPFLLRNGANEGF
401
HEAVGEIMSLSAATPKHLKSIGLLSPDFQEDNETEINFLLKQALTIVGTL
73.6
18.9
27.3
451
PFTYMLEKWRWMVFKGEIPKDQWMKKWWEMKREIVGVVEPVPHDETYCDP
61.9
52.6
40.2
31.9
58.9
24.0
501
ASLFHVSNDYSFIRYYTRTLYQFQFQEALCQAAKHEGPLHKCDISNSTEA
20.5
551
GQKLFNMLRLGKSEPWTLALENVVGAKNMNVRPLLNYFEPLFTWLKDQNK
54.5
46.0
601
NSFVGWSTDWSPYADQSIKVRISLKSALGDKAYEWNDNEMYLFRSSVAYA
58.7
11.1
651
MRQYFLKVKNQMILFGEEDVRVANLKPRISFNFFVTAPKNVSDIIPRTEV
58.7
26.4
18.9
26.7
63.9
26.9
701
EKAIRMSRSRINDAFRLNDNSLEFLGIQPTLGPPNQPPVSIWLIVFGVVM
26.7
4.7
26.9

751

GVIVVGIVILIFTGIRDRKKKNKARSGENPYASIDISKGENNPGFQNTDD

45.2

46.4

16.6

801

VQTSF

46.4

* indicates peptide not unique

Color code for signal intensity

>6.8e+006	>2.0e+006	>6.1e+005	>1.8e+005	>5.4e+004

Sequence Coverage Map ACE2 in EndoC-βH1 (Exp2)

Minimum MS Signal = 20000
Protease = Trypsin

Proteins	Number of MS Peaks	MS Peak Area	Sequence Coverage	Abundance (mol)
1:ACE2	88	0.3%	36.1%	100.00%
Unidentified	43608	99.7%		

MS Signal Threshold = 3.3e+004

ACE2

1–50
MSSSSWLLLSLVAVTAAQSTIEEQAKTFLDKFNHEAEDLFYQSSLASWNY
4.5

51–100
NTNITEENVQNMNNAGDKWSAFLKEQSTLAQMYPLQEIQNLTVKLQLQAL

101–150
QQNGSSVLSEDKSKRLNTILNTMSTIYSTGKVCNPDNPQECLLLEPGLNE
43.0
23.9

151–200
IMANSLDYNERLWAWESWRSEVGKQLRPLYEEYVVLKNEMARANHYEDYG
3.5
33.9

201–250
DYWRGDYEVNGVDGYDYSRGQLIEDVEHTFEEIKPLYEHLHAYVRAKLMN
5.7
45.5
26.6
32.9
51.6

251–300
AYPSYISPIGCLPAHLLGDMWGRFWTNLYSLTVPFGQKPNIDVTDAMVDQ
45.5
26.9
26.6
32.9

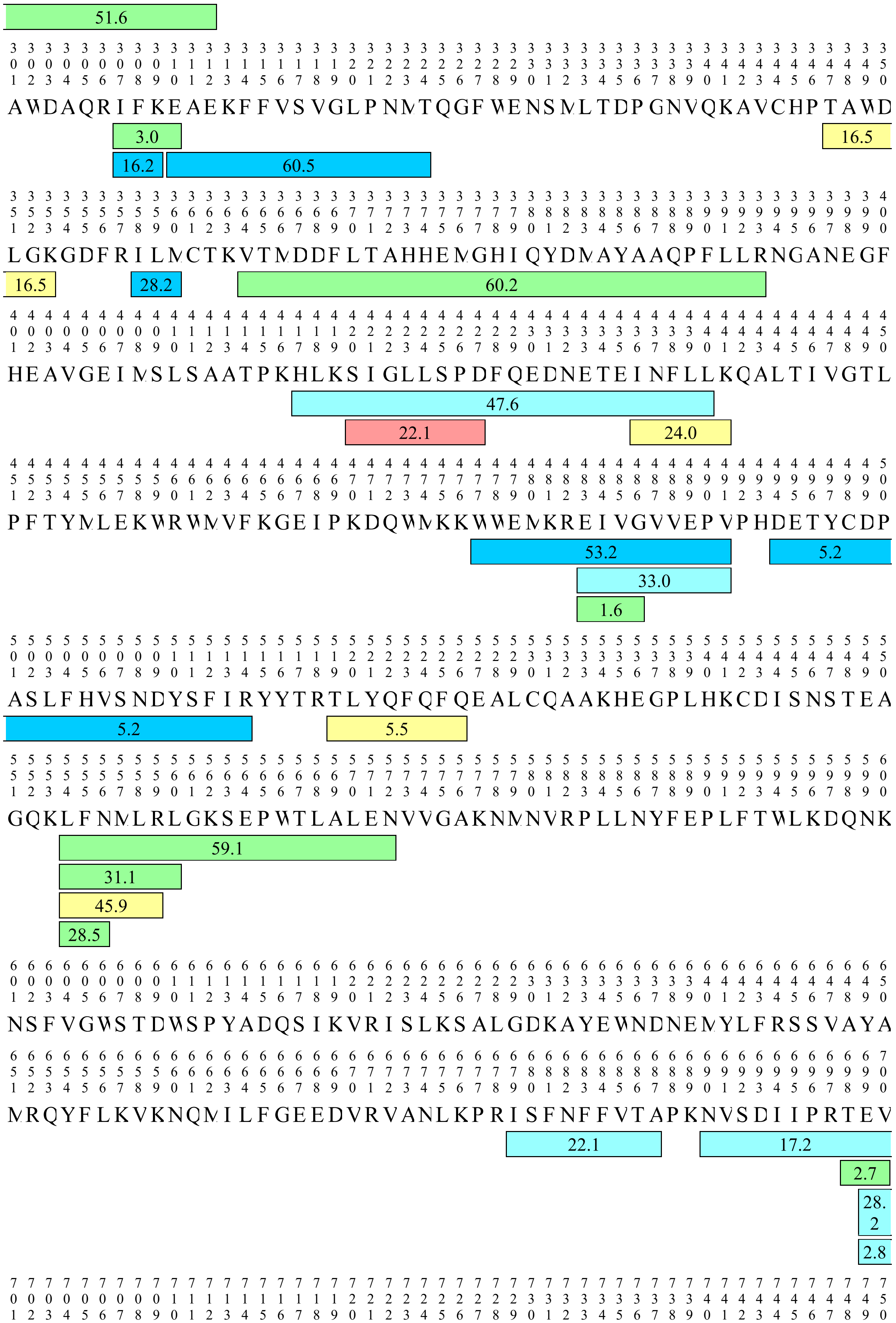
51.6
AWDAQRIFKEAEKFFVSVGLPNMTQGFWENSMLTDPGNVQKAVCHPTAWD
3.0
16.5
16.2
60.5
LGKGDFRILMCTKVTMDDFLTAHHEMGHIQYDMAYAAQPFLLRNGANEGF
16.5
28.2
60.2
HEAVGEIMSLSAATPKHLKSIGLLSPDFQEDNETEINFLLKQALTIVGTL
47.6
22.1
24.0
PFTYMLEKWRWMVFKGEIPKDQWMKKWWEMKREIVGVVEPVPHDETYCDP
53.2
5.2
33.0
1.6
ASLFHVSNDYSFIRYYTRTLYQFQFQEALCQAAKHEGPLHKCDISNSTEA
5.2
5.5
GQKLFNMLRLGKSEPWTLALENVVGAKNMNVRPLLNYFEPLFTWLKDQNK
59.1
31.1
45.9
28.5
NSFVGWSTDWSPYADQSIKVRISLKSALGDKAYEWNDNEMYLFRSSVAYA
MRQYFLKVKNQMILFGEEDVRVANLKPRISFNFFVTAPKNVSDIIPRTEV
22.1
17.2
2.7
28.2
2.8

EKAIRMSRSRINDAFRLNDNSLEFLGIQPTLGPPNQPPVSIWLIVFGVVM

17.2

28.2

2.8

751 752 753 754 755 756 757 758 759 760 761 762 763 764 765 766 767 768 769 770 771 772 773 774 775 776 777 778 779 780 781 782 783 784 785 786 787 788 789 790 791 792 793 794 795 796 797 798 799 800

GVIVVGIVILIFTGIRDRKKKNKARSGENPYASIDISKGENNPGFQNTDD

63.4

3.2

47.1

20.9

801 802 803 804 805

VQTSF

47.1

Color code for signal intensity

>3.3e+006	>1.0e+006	>3.3e+005	>1.0e+005	>3.3e+004
